# Atlanta Medical College—Failure of Its Enemies

**Published:** 1871-06

**Authors:** 


### Editorial and Miscellaneous.

*Atlanta Medical College—The Failure of its Enemies.*

In our last issue we gave a faithful history of the attacks made upon the College, and the motives which probably actuated the leaders. This "statement of facts" had been deferred for years, with the hope that persecutors of the institution would be able some time to satiate their thirst for revenge, and save us the unpleasantness of having to array them before the public in their true character. This forbearance, however, only served to increase their efforts, until "patience ceased to be a virtue," and the Faculty, with other friends of justice, propriety and common sense, determined to meet the perpetrators of the assaults at the threshhold, in every attempt to traduce the institution. With this view, physicians opposed to the further use of the Georgia Medical Association, for this purpose, attended the last meeting in Americus, and put the seal of condemnation upon such prostitution of an association, designed for the advancement of science and good will (not strife) in the medical profession of the State. This was done by a very decided majority (two-thirds) of members present. Chagrined at failing in their cherished idea of running the Association to the disparagement of the Atlanta College, the malcontents, consisting chiefly of the nucleus in Atlanta, the Faculty and other friends of Savannah College, and the friends and expectants, probably, of a contemplated college in Macon, called a Convention of the physicians of the State in order to perpetuate strife to the detriment of the College in Atlanta, under the guise of having action which may "guide the Association" at its next meeting in Columbus in making a "final settlement of this College trouble."

This explanation will account not only for the course of physicians in Macon, but for that of some who *may* be aspirants outside.

That these are the motives which prompt our persecutors, we find proof in the fact that conciliatory acts on the part of those traduced serve only to increase the efforts of their pursuers. A feature in the College charter, giving the Faculty power to fix terms for the graduation of students, having been exercised eight years without objection, was made the point for the first *public* attack. This power is possessed *absolutely* by Faculties of Nashville, Louisville, and other Southern and Western Colleges, to say nothing of Augusta, and other prominent Colleges, whose Faculties *virtually*, and properly so, control such matters. That feature was, however, conceded by the Faculty for the sake of peace, but with no other effect than to encourage the assailants. Apologies are demanded, but nothing proves satisfactory.

The Convention met, and finding no College trouble with the Association to settle, adjourned, without any action to the disparagement of individuals or institutions. Every well meaning man is compelled to feel in his heart that the Convention did right, and the action had by it, and by the State Association at Americus, is a withering rebuke to those who seek to gratify revenge or jealousy by assaults on individuals or institutions through the State Medical Association.

Toward the malcontents we indulge no feeling of animosity, and seek the adoption of no measure detrimental to them, individually or professionally. We oppose only those acts which tend to the injury of honorable members of the profession.

While we would advise honorable submission to the will of the State Association, and to the Convention of their own calling, we will exercise the charity to allow, unrebuked, an outburst of anger in the denial of a "true statement of facts" in history and the indulgence of unbecoming epithets toward members of the Convention through the Macon press. All the members were approved by a committee appointed *very early* in the session by their own organization. The truth is, men should guard well against ebulition of temper "when whipped at their own game;" and should never deny in fierce and angry terms that which everybody knows to be true.

If other institutions, or those not connected with colleges, see proper to make themselves judges of the propriety of Atlanta Medical College relieving herself of disagreeable members of the

Faculty, let them do so openly and on a true issue. To this we cannot object, nor would any one connected with the institution here, we presume, object to any other college taking such members into their own faculty, should sympathy and good feeling extend so far toward them.

Conciliatory measures and concessions on true points of issue generally lead to reconciliation of honorable parties at variance, but men bent on mischief, making unjust demands, are never appeased by having them granted. Concessions to men thus inclined serve to make them persevere in and multiply their demands. We presume that no one will be again deceived into the opinion that peace, short of restoring expelled members of the Faculty, or the downfall of the College here, will be acceptable to some of our assailants. The former, the Trustees have already refused to do, and to effect the latter, hundreds of such unjust attacks as that made through the *secular* paper in Macon will not be sufficient. We have good authority for stating that the expelled member, who inaugurated the opposition, proposes that attacks shall cease on condition he can be replaced in the College only for one day; proving that all ethical and other ostensible objections are subordinate to this personal consideration.

We have now concluded what we have to say, in the Journal, upon this opposition to the College. It is with reluctance we commenced it in the last number, knowing that already the subject had been too often thrust upon the public. We, however, supposed that a full and fair statement of the origin of strife should be made in justice to the College and to the profession. We have now done so, and shall not again intrude this subject upon our readers.

